# Phospholipase PlcH is involved in the secretion of cell wall glycoproteins and contributes to the host immune response of *Aspergillus fumigatus*

**DOI:** 10.1002/mlf2.12146

**Published:** 2024-12-26

**Authors:** Jinbin Hao, Yin Guo, Hui Zhou, Haomiao Ouyang, Jinghua Yang, Wenxia Fang, Cheng Jin

**Affiliations:** ^1^ State Key Laboratory of Mycology, Institute of Microbiology Chinese Academy of Sciences Beijing China; ^2^ University of Chinese Academy of Sciences Beijing China; ^3^ Colleg of Life Science and Technology Guangxi University Nanning China; ^4^ Institute of Biological Sciences and Technology Guangxi Academy of Sciences Nanning China

**Keywords:** *Aspergillus fumigatus*, cell wall protein, GPI‐anchored protein, immune response, macrophage killing

## Abstract

Glycosylphosphatidylinositol (GPI) anchoring is one of the conserved posttranslational modifications in eukaryotes that attach proteins to the plasma membrane. In fungi, in addition to plasma membrane GPI‐anchored proteins (GPI‐APs), some GPI‐APs are specifically released from the cell membrane, secreted into the cell wall, and covalently linked to cell wall glucans as GPI‐anchored cell wall proteins (GPI‐CWPs). However, it remains unclear how fungal cells specifically release GPI‐CWPs from their membranes. In this study, phospholipase PlcH was identified and confirmed as a phospholipase C that hydrolyzes phosphate ester bonds to release GPI‐APs from the membrane of the opportunistic fungal pathogen *Aspergillus fumigatus*. Deletion of the *plcH* gene led to abnormal conidiation, polar abnormality, and increased sensitivity to antifungal drugs. In an immunocompromised mouse model, the Δ*plcH* mutant showed an attenuated inflammatory response and increased macrophage killing compared with the wild type. Biochemical and proteomic analyses revealed that PlcH was involved in the localization of various cell wall GPI‐APs and contributed to the cell wall integrity. Our results demonstrate that PlcH can specifically recognize and release GPI‐CWPs from the cell membrane, which represents a newly discovered secretory pathway of GPI‐CWPs in *A. fumigatus*.

## Impact statement

*Aspergillus fumiatus* is a fungal pathogen causing life‐threatening infection. As one of the cell wall components, glycoproteins form the outer layer and protect *A. fumigatus* from the immune attack of the host. However, less is known about how these glycoproteins are secreted into the cell wall. Here, we identify phospholipase PlcH as the enzyme to release one set of cell wall glycoproteins from the plasma membrane of *A. fumigatus*. Cell wall glycoproteins released by PlcH contribute to the cell wall integrity and immune response. Our findings provide a novel insight into the complicated mechanism of fungal cell wall assembly.

## INTRODUCTION

*Aspergillus fumigatus* is the most common *Aspergillus* species causing invasive aspergillosis (IA). IA has become a severe health problem, with a mortality rate higher than 50% and reaching 95% in certain situations^1^, ^2^, ^3^, ^4^, and has been reported to coinfect with severe influenza or COVID‐19^2^, ^5^. The high mortality rate is due to limited antifungal drugs and the continuous increase in drug‐tolerant strains. New drugs are urgently needed^6^, ^7^, ^8^.

As the cell wall is essential for the growth of fungi and does not exist in humans or animals, it has been identified as an important target for the development of antifungal drugs^9^, ^10^, ^11^, ^12^. The fungal cell wall is mainly composed of chitin, glucans, and glycoproteins (also known as cell wall mannoproteins). The synthesis and secretion of cell wall polysaccharides have been extensively studied^13^, ^14^, ^15^. Glycoproteins form a network in the outermost layer of the cell wall, which forms the first barrier that comes into contact with the environment and is essential for fungal survival^16^. However, compared with cell wall polysaccharides, it remains unclear how glycoproteins secrete and localize in the cell wall.

Glycosylphosphotidylinositol (GPI) anchoring is one of the posttranslational modifications conserved in eukaryotes from yeast to humans. When the C‐terminus of a protein harbors a GPI signal, the GPI signal of the protein is excised (the excision site is called the ω site), and the newly exposed C‐terminus is covalently linked to the GPI anchor, which anchors the protein in the outer leaflet of the plasma membrane to become a membrane‐bound GPI‐anchored protein (GPI‐AP). In mammalian cells, some GPI‐APs are released from the membrane by GPI phospholipases, such as GPI‐PLD, angiotensin‐converting enzyme, testicular angiotensin‐converting enzyme, glycerophosphate dilipase 2, and phospholipase A2 (PGAP6/TMEM8A). GPI‐APs released by these specific enzymes are involved in the regulation of blood pressure, spermatogenesis, and neurogenesis^17^, ^18^. Unlike mammalian cells, fungal cells can specifically secrete some GPI‐APs from the cell membrane into the cell wall to become GPI‐anchored cell wall proteins (GPI‐CWPs) via the covalent link to cell wall glucans^16^. A number of *A. fumigatus* cell wall glycoproteins are GPI‐CWPs^19^, ^20^, which are essential for cell wall integrity and virulence^21^, ^22^, ^23^, ^24^. However, the mechanism of the secretion of these cell wall glycoproteins remains unknown.

Generally, it is believed that GPI‐CWPs are secreted by hydrolyzing the glycosidic bond between GlcN and Man1 in the GPI anchor, and then, Man1 is attached to the cell wall β1, 6‐glucan^16^. Although the full process and mechanism are still unknown, investigations of *Saccharomyces cerevisiae* and *Candida albicans* suggest that two GH76 family homologs, Dfg5 and Dcw1, may be involved in the hydrolysis of the glycosidic link between Manα1,4‐GlcN in the GPI core sugar chain and covalently link the protein to β1, 6‐glucan^25^. When Dcw1 and Dfg5 are deleted, the yeast cell wall is defective and GPI‐CWPs are secreted into the medium^26^, ^27^, ^28^, suggesting that Dfg5 and Dcw1 are associated with the localization of GPI‐CWPs in the cell wall. Nine members of the GH76 family have been identified in *Neurospora crassa*, of which two are involved in cell wall synthesis by cleaving N‐linked mannan and then transporting glycoprotein to the cell wall. Eight members of the GH76 family have been found in *A. fumigatus*, of which six are not essential, and the loss of these genes results in a failure of translocation of GPI‐anchored galactomannan to the cell wall. However, they are not involved in the secretion of GPI‐CWPs^25^. It is likely that, in *A. fumigatus*, GPI‐CWPs are secreted through a pathway different from that in yeasts.

In this study, *A. fumigatus AFUA_4G12000* was identified as the gene encoding a putative phospholipase, namely, PlcH. Using biochemical, genetic, and proteomic approaches, we confirmed that PlcH is a GPI‐phospholipase C responsible for the release of GPI‐CWPs from the plasma membrane of *A. fumigatus*. Deletion of the *plcH* gene led to a cell wall defect, earlier germination, polar abnormality, and reduced conidiation. In addition, increased drug sensitivity and reduced immune response were documented in the deletion mutant.

## RESULTS

### PlcH is a phospholipase C that releases GPI‐CWPs from the plasma membrane

BLASTP searching of the *A. fumigatus* Af293 genome database^29^ with PIPLC protein sequences of *Candida orthopsilosis* (XP_003866914.1), *Candida albicans* (CAB92911.1), *Bacillus thuringiensis* (WP_134996341.1), and *Staphylococcus aureus* (CAQ48551.1) identified *AFUA_4G12000* as the gene encoding a putative phosphatidylinositol phospholipase C (PIPLC), which is 1434 bp in length and renamed *plcH*. The *plcH* gene encodes a 55‐kDa protein that shares a high homology with PIPLC. The catalytic centers of prokaryotic and eukaryotic PIPLCs contain two catalytic histidines. Compared with other homologous proteins, PlcH also contains the conserved amino acid residues His‐172 and His‐223 (Figure S1A), and the tertiary structure prediction suggests that these two conserved histidines are distributed in the active pockets of proteins (Figure S1B). In fungi, PIPLC homologs mainly contain two active domains, X and Y^30^, and can be divided into two groups. Group 1 contains only the X domain, whereas Group 2 contains both the X and Y domains. PlcH belongs to Group 1 and is similar to bacterial PIPLC (Figure S1C).

**Figure 1:**
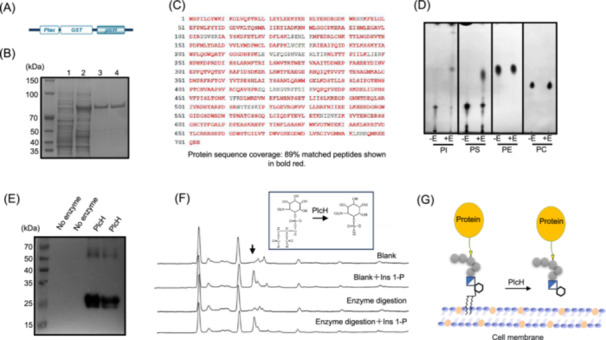
Expression, activity assay, and substrate specificity of recombinant PlcH. (A) Schematic diagram of the expression vector. The PGEX‐6P1‐PlcH vector carrying *plcH* cDNA was used to express the GST‐PlcH fusion protein. (B) Purification of recombinant PlcH. GST‐PlcH was purified using GST‐tag purification resin (Beyotime), and the purified protein was separated using 12% SDS‐PAGE. Lane 1, an intracellular protein expressed by the *Escherichia coli* BL21(DE3)pLysS harboring PGEX‐6P1 vector, which was used as a control; lane 2, an intracellular protein expressed by the *E. coli* BL21(DE3) pLysS harboring GEX‐6P1‐PlcH vector; and lanes 3 and 4, purified GST‐PlcH. (C) Confirmation of purified recombinant PlcH by LC‐MS/MS. Purified GST‐PlcH was confirmed using LC‐MS/MS, matched peptides are shown in bold red, and the sequence coverage was 89%. (D) Thin layer chromatography (TLC) analysis of the hydrolytic products of recombinant PlcH. A hospholipase assay was conducted using phosphatidylcholine (PC), phosphatidylethanolamine (PE), phosphatidylserine (PS), and phosphatidylinositol (PI) as substrates and the products were analyzed with TLC. PI and PS can be degraded by purified PlcH. (E) Detection of Mp1 released by PlcH. The membrane fraction was extracted as described in the Materials and Methods section and incubated at 30°C for 1 h with or without purified PlcH. After centrifugation, Mp1 protein was detected in the supernatant by immunoblotting with anti‐Mp1 monoclonal antibody. (F) Identification of the cleavage site of PlcH using HPLC. Blank, the PLC buffer was utilized to replace PlcH in the experiment as a negative control; blank + Ins 1‐P, the supernatant of the blank group was supplemented with the internal standard Ins 1‐P for determining the internal standard peak of HPLC; enzyme digestion, the released glycosylphosphatidylinositol (GPI) proteins from the membrane pellets by PlcH were treated with a weak base followed by a weak acid to release inositol 1‐phosphate (Ins 1‐P); enzyme digestion + Ins 1‐P, the supernatant of the enzyme digestion group was supplemented with the internal standard Ins 1‐P to identify the internal standard peak in HPLC. (G) Model of the GPI‐APs' release from the plasma membrane by PlcH. GPI‐AP, glycosylphosphatidylinositol‐anchored protein; LC‐MS/MS, liquid chromatography‐tandem mass spectrometry.

To determine the enzymatic activity of PlcH, the *plcH* gene was inserted into a pGEX‐6P‐1 vector carrying glutathione S‐transferase (GST) and transformed into *E. coli* BL21(DE3)pLysS (Figure 1A). The GST‐PlcH fusion protein was purified (Figure 1B). Liquid chromatography‐tandem mass spectrometry (LC‐MS/MS) analysis revealed that the sequence coverage of purified GST‐PlcH was 89% (Figure 1C). Using phosphatidylcholine (PC), phosphatidylethanolamine (PE), phosphatidylinositol (PI), and phosphatidylserine (PS) as substrates, the activity of purified PlcH was assayed. As shown in Figure 1D, PlcH was able to hydrolyze PS and PI specifically and released free fatty acids. These results confirm that PlcH possesses phospholipase C activity toward PS and PI.

To test whether PlcH can release GPI‐CWPs from the plasma membrane, *A. fumigatus* membrane components were extracted. Purified PlcH was incubated with the membrane fraction at 30°C for 1 h. After centrifugation, the supernatant was detected with an anti‐Mp1 antibody. As shown in Figure 1E, specific GPI‐CWP Mp1 was detected, indicating that PlcH is able to release GPI‐CWPs from the membrane. To further elucidate the cleave site of PlcH, we analyzed the product of PlcH. After the removal of the inositol‐attached fatty acid chain with ammonia water and the release of inositol 1‐phosphate with acetic acid, the PlcH‐hydrolyzed product was detected using high‐performance liquid chromatography (HPLC). Inositol 1‐phosphate was detected in the product derivatives (Figure 1F), indicating that PlcH can cleave the phosphate ester bond between inositol and phosphate (Figure 1G). These results demonstrate that PlcH hydrolyzes the phosphate ester bond between inositol and the fatty acid chain to release GPI‐APs from the plasma membrane.

### PlcH is associated with the secretion and localization of GPI‐CWPs

**Figure 2:**
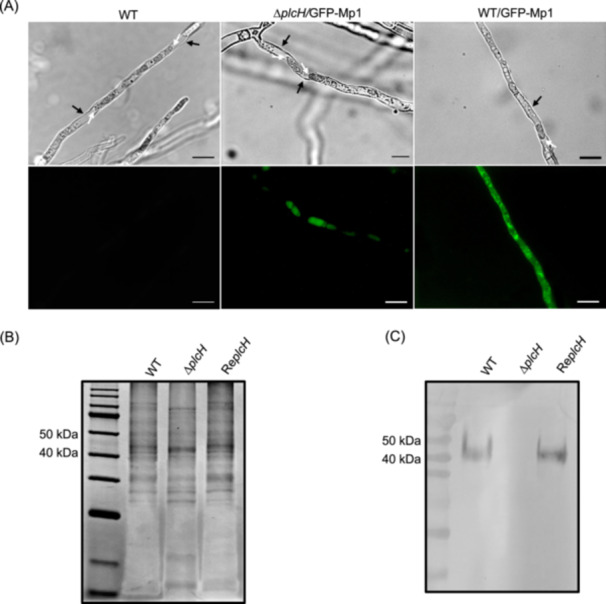
Localization of GPI‐APs in the cell wall of the Δ*plcH* mutant. (A) Localization of chimeric GFP‐Mp1 in the wild‐type (WT) and the Δ*plcH* mutant strain. To track the localization of the Mp1 protein, the vector pGFP/Mp1 harboring the gene encoding GFP‐Mp1 was introduced into the WT or the Δ*plcH* mutant. After cultivation on a solid complete medium (CM) at 37°C for 48 h, the mycelia were subjected to plasmolysis with 0.5 M sorbitol and observed under fluorescence microscopy (Leica Microsystems). After plasmolysis, fluorescence was concentrated in the shrunken cells of the Δ*plcH* mutant expressing GFP‐Mp1 (Δ*plcH*/GFP‐Mp1), while fluorescence was detected in the shrunken cells and cell walls of the WT expressing GFP‐Mp1 (WT/GFP‐Mp1). (B) SDS‐PAGE of the cell wall proteins of the WT, mutant, and revertant strain. (C) Western blot analysis of cell wall Mp1 protein. Western blot analysis was performed with an anti‐Mp1 monoclonal antibody as described in the Materials and Methods section. No Mp1 was detected in the cell wall of the mutant compared with that of the WT or revertant. The cell wall and the cell membrane are marked by black arrows and white arrows, respectively. The scale bar indicates 20 μm.

To evaluate the role of PlcH in the secretion of GPI‐CWPs in *A. fumigatus*, the *plcH* gene was knocked out, and the Δ*plcH* mutant was confirmed by Southern blot and PCR (Figure S2). Previously, we showed that the C‐terminal sequence of Mp1 (48 amino acid residues containing the region from ω‐23 to ω‐1 and the GPI signal sequence) fused to the C‐terminal of green fluorescent proteins (GFP) directed the localization of GFP‐Mp1 in the cell wall of *A. fumigatus* ^31^. To track the subcellular localization of GPI‐CWPs in the mutant, the chimeric protein GFP‐Mp1, a GFP protein fusion with the C‐terminal sequence of Mp1 at its C‐terminal, was expressed in either the wild‐type (WT) or Δ*plcH* mutant. The treatment of mycelia with 0.5 M sorbitol resulted in shrinkage of the mycelial cell and thus separation of the plasma membrane from the cell wall^31^. Using this strategy, the location of chimeric GFP‐Mp1 can be visualized under fluorescence microscopy. As a result, GFP‐Mp1 was found in the cell wall of the WT, while GFP‐Mp1 was bound to the membrane of the Δ*plcH* mutant (Figure 2A). The Mp1 protein was not detected in the mutant cell wall by immunoblotting with an anti‐Mp1 monoclonal antibody (Figure 2B,C). These results confirm that PlcH is responsible for the release of GPI‐CWPs such as Mp1.

**Figure 3:**
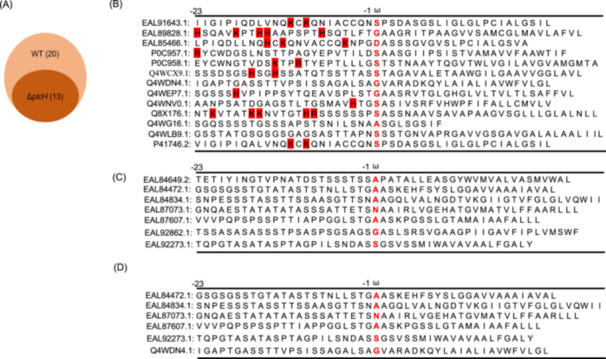
Analysis of GPI‐CWPs released by PlcH. (A) Venn diagram showing a difference in GPI‐CWPs between the WT and the Δ*plcH* mutant. (B) Amino acid sequence of common GPI‐CWPs between the WT and the Δ*plcH* mutant. (C) Amino acid sequence of the missing GPI‐CWPs in the cell wall of the Δ*plcH* mutant. (D) Amino acid sequence of GPI‐CWPs released from the mutant cell membrane by purified PlcH.

Cell wall proteins were extracted from the WT and the Δ*plcH* mutant, separated with SDS‐PAGE, and analyzed using LC‐MS/MS. Combined with GPI‐AP prediction (https://mendel.imp.ac.at/gpi/fungi) and subcellular localization prediction (https://wwwgenscript.com/wolf-psort.html), 20 proteins were identified as GPI‐CWPs in the WT. Most have not been studied previously. Thirteen GPI‐CWPs were found in the WT and the Δ*plcH* mutant (Table S1), suggesting that PlcH does not impede the cell wall localization of these proteins (Figure 3A). Compared with the WT, seven GPI‐CWPs were missing in the mutant cell wall (Table S2), including Mp1 and Mp2, indicating that PlcH is involved in the release of these GPI‐CWPs from the membrane.

Interestingly, we found that 10 of the 13 retained GPI‐CWPs in the mutant cell wall contained basic amino acids in the region from ω‐1 to ω‐23 (Figure 3B and Table S1). The seven GPI‐CWPs that disappeared in the mutant did not contain any basic amino acids in this region (Figure 3C and Table S2). These observations suggest that PlcH can cleave GPI‐APs without any basic amino acid residue in the region from ω‐1 to ω‐23. It is likely that GPI‐CWPs containing basic amino acids in the region from ω‐1 to ω‐23 might be released by other enzymes instead of PlcH. To verify this hypothesis, the membrane components of the mutant were extracted and incubated with recombinant PlcH. GPI‐APs released from the membrane were analyzed using MS. As shown in Figure 3D and Table S3, six GPI‐CWPs were released from the membrane by recombinant PlcH, including EAL84472.1 (Mp1), EAL87607.1 (Mp2), Q4WDN4.1 (OpsB), EAL92273.1, EAL87073.1, and EAL84834.1. No basic amino acid was found in the region from ω‐1 to ω‐23 of these proteins, confirming that PlcH can cleave GPI proteins without any basic amino acid residue in the region from ω‐1 to ω‐23.

### Deletion of the *plcH* gene results in a cell wall integrity defect

**Figure 4:**
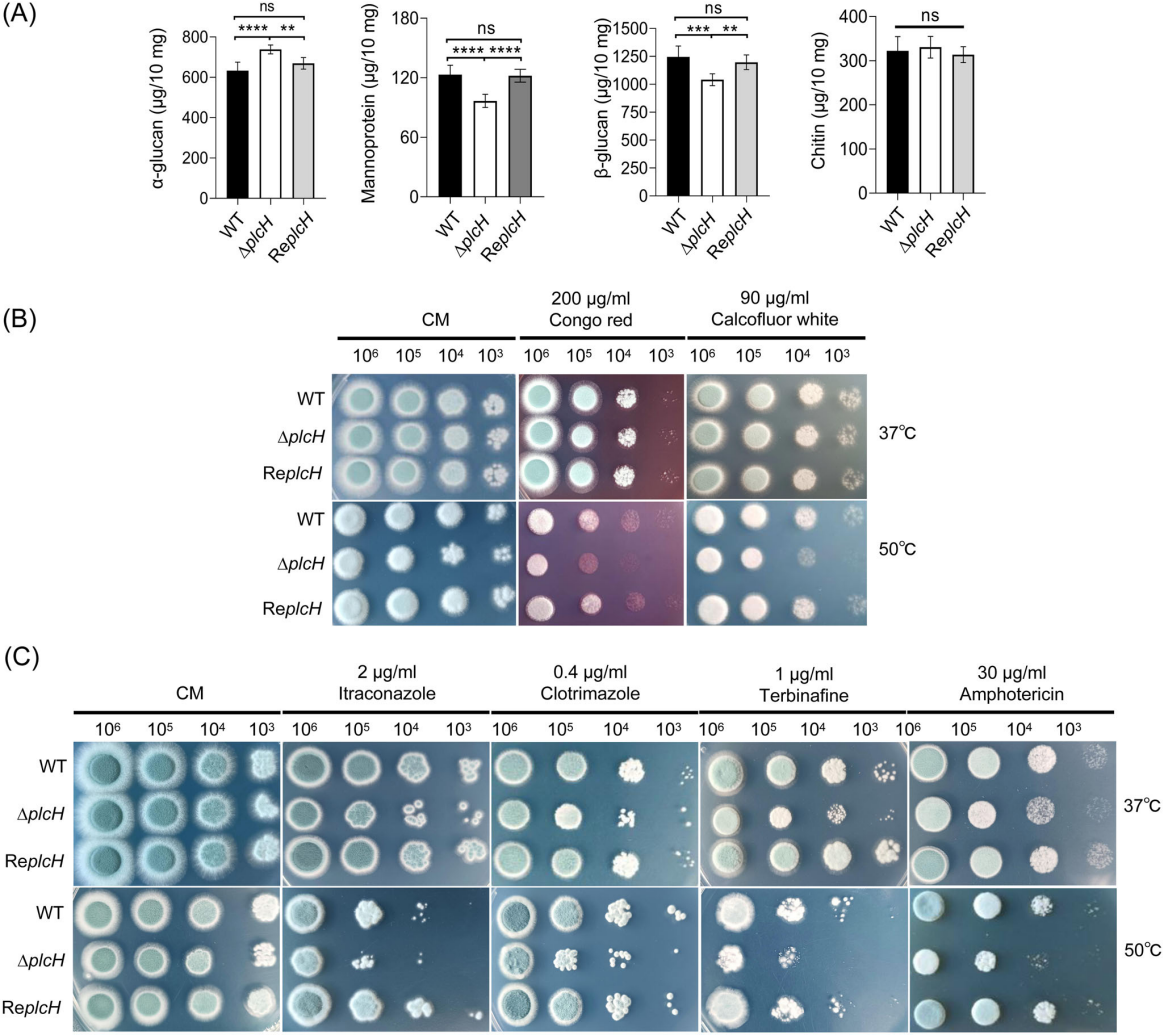
Analyses of cell wall contents and drug sensitivity of the Δ*plcH* mutant. (A) Chemical analysis of the mutant cell wall. A total of 10^6^ conidia were inoculated into 100 ml liquid CM and incubated at 37°C. Three 10 mg aliquots of dried mycelium were used as independent samples for cell wall analysis according to the procedures described in the Materials and Methods section. The experiment was repeated three times. The numbers are expressed as micrograms of cell wall components per 10 mg of dry mycelium. The asterisks show statistically significant differences (ns, not significant; **p < 0.01; ***p < 0.001; ****p < 0.0001) based on one‐way ANOVA (WT vs. Δ*plcH*, WT vs. Re*plcH*, and Δ*plcH* vs. Re*plcH*). (B) Sensitivity of the mutant to Calcofluor white and Congo red. A serial dilution of conidia from 10^6^ to 10^3^ was spotted on solid CM with a cell wall synthesis disruptor (Congo red and Calcofluor white) and incubated at 37°C and 50°C for 48 h. (C) Sensitivity of the mutant to various antifungal drugs. A serial dilution of conidia from 10^6^ to 10^3^ was spotted on solid CM with antifungal drugs (Itraconazole, Clotrimazole, Terbinafine, and Amphotericin) and incubated at 37°C and 50°C for 48 h.

To evaluate the influence of PlcH on cell wall integrity, the cell wall contents of the mutant were measured. As shown in Figure 4A, the mannoprotein and β‐glucan contents of the mutant grown at 37°C were significantly lower than those of the WT or revertant strain. While the α‐glucan content was significantly increased and the chitin content remained unchanged. These results demonstrate that *plcH* deletion leads to reduced mannoproteins and β‐glucans in the cell wall.

We further examined the sensitivities of the mutant to various cell wall‐disturbing agents and antifungal drugs. Calcofluor white and Congo red affect cell walls by interfering with the biosynthesis of cell wall chitin and glucan, respectively^32^, ^33^, ^34^. The sensitivity to Calcofluor white and Congo red of the mutant Δ*plcH* was slightly increased at 50°C (Figure 4B), indicating that deletion of the *plcH* gene interferes with cell wall biosynthesis at a higher temperature and that the increase in α‐glucans can compensate for the cell wall defect at 37°C (Figure 4A). Itraconazole and Clotrimazole are azole drugs that block the biosynthesis of ergosterol by targeting cytochrome P450‐dependent enzymes^35^, ^36^, ^37^, ^38^. Terbinafine can inhibit squalene epoxidase and interfere with the biosynthesis of ergosterol^39^, ^40^. Amphotericin can form a complex with ergosterol and disrupt the fungal cell membrane^41^, ^42^. In comparison to the WT or revertant strain, the Δ*plcH* mutant showed slightly increased sensitivity to Itraconazole, Clotrimazole, Terbinafine, and Amphotericin at 37°C and 50°C (Figure 4C).

**Figure 5:**
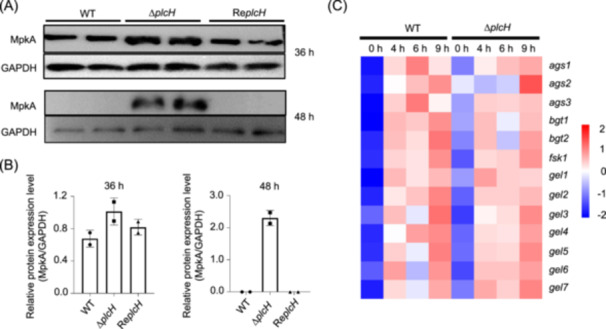
Activation of MpkA and expression of the genes involved in cell wall synthesis in the Δ*plcH* mutant. (A) Phosphorylation of MpkA induced by deletion of the *plcH* gene. The conidia were inoculated in liquid CM and incubated at 37°C for 36 h or 48 h. Phosphorylated MpkA was detected by western blot analysis using anti‐Phospho‐p44/42 MAPK. GAPDH was used as control. (B) Phosphorylation level of MpkA. The phosphorylated MpkA content was measured and calculated using ImageJ. (C) The expression of genes related to cell wall polysaccharide synthesis in the Δ*plcH* mutant at different stages (0, 4, 6, and 9 h) analyzed by qRT‐PCR and visualized by a heat map.

In *A. fumigatus*, MpkA is a central regulator in the cell wall integrity (CWI) signaling pathway^22^. In the mutant, the phosphorylation level of MpkA was found to be dramatically increased compared with that of the WT and the revertant strain (Figure 5A,B), indicating the activation of the mutant's CWI signaling pathway. Additionally, the expression of the genes required for the synthesis of cell wall polysaccharide in the Δ*plcH* mutant was examined at the conidia germination stage (Figure 5C). After 4 h of cultivation, the α‐glucan synthesis‐related genes *ags1* and *ags2* and the β‐glucan synthesis‐related genes *gel3* and *gel6* were downregulated compared to WT (Figures 5C and S3A). Expression of *ags3*, *bgt1*, *bgt2*, and *gel4* was also downregulated when the conidia were cultured for 6 h, and expression of *fsk1*, *gel1*, *gel3*, and *gel5* was upregulated (Figures 5C and S3B). When the culture time reached 9 h, expression of the β‐glucan synthesis‐related genes *bgt1*, *bgt2*, *gel1*, *gel2*, and *gel3* was downregulated, and expression of α‐glucan synthesis related genes *ags1*, *ags2*, and *ags3* was upregulated (Figures 5C and S1C). These results indicate that the loss of PlcH triggers a complicated compensation of cell wall synthesis.

### Deletion of the *plcH* gene leads to abnormal polarity and a defective conidial cell wall

**Figure 6:**
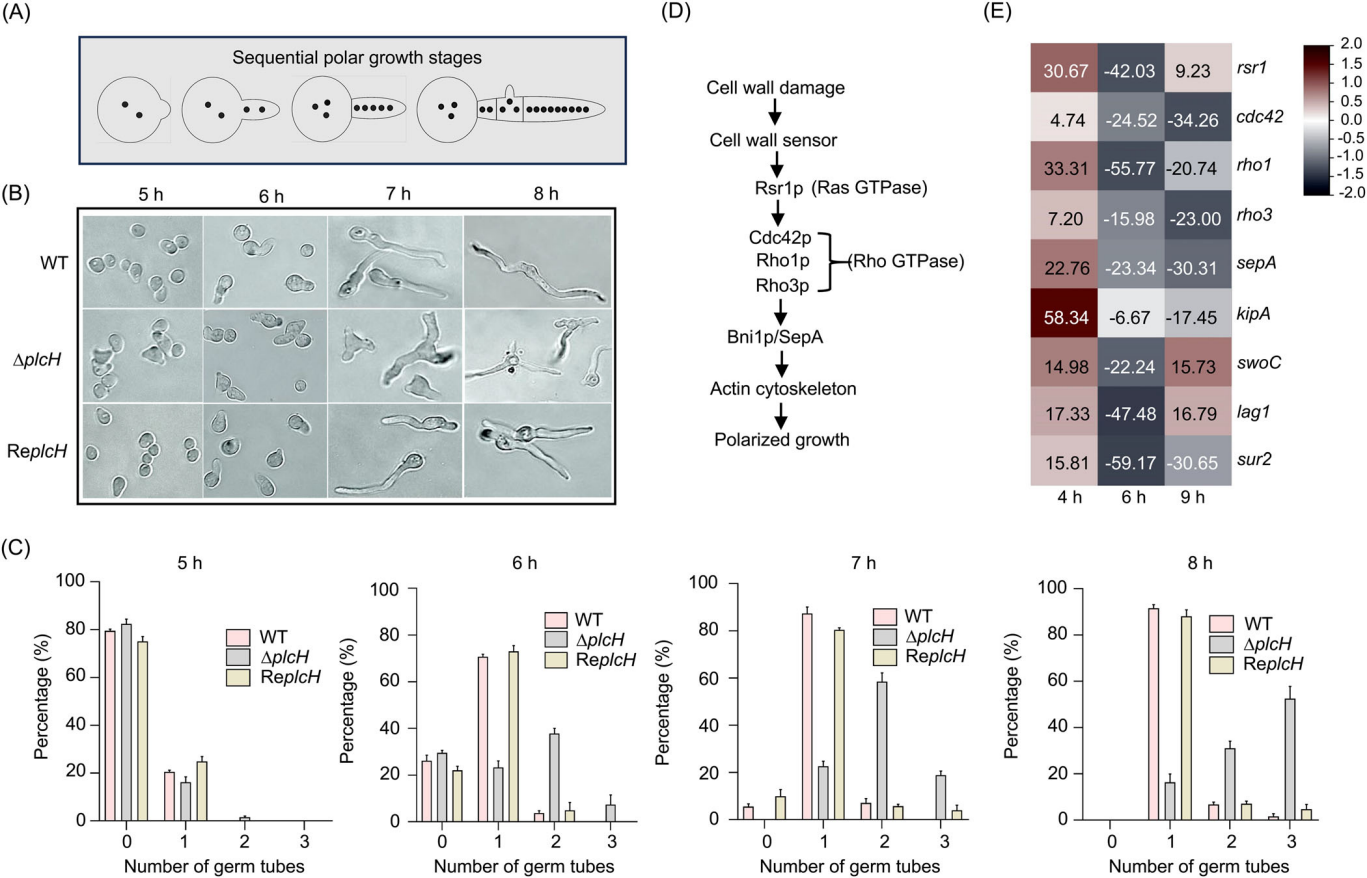
Conidia germination of the Δ*plcH* mutant. (A) Schematic diagram of sequential polar growth of *Aspergillus fumigatus*. (B) Germination of the mutant conidia. A total of 10^6^ freshly washed spores were inoculated on Petri dishes containing 10 ml liquid CM with sterilized glass slides and incubated at 37°C. The coverslips with germination conidia were taken out, fixed in paraformaldehyde (PFA) solution (50 mM pH7.0 phosphate buffer containing 3.7% paraformaldehyde and 0.1% Triton X‐100), washed several times with phosphate‐buffered saline (PBS) and observed. (C) Counting of germ tubes at the germination stage under a differential interference contrast microscope. One hundred cells were counted, and this was repeated three times. The values shown are the mean ± SD. (D) Depiction of the signaling pathway involved in polarity in fungi based on references^43^, ^44^, ^45^, ^46^. (E) The expression of polar‐related key genes at different stages (4, 6, and 9 h) of conidial germination was visualized with heat maps.

Normally, *A. fumigatus* conidia germinate in a bipolar pattern, typically at an angle of 180°. After four rounds of mitosis (7–8 h), the second germ tube and the first septation are formed. As can be seen in Figure 6A–C, the Δ*plcH* mutant conidia germinated earlier than that of the WT or revertant. Around 1.5% of the mutant conidia germinated the second germ tube at an abnormal angle to the first germ tube at 5 h, 7.4% germinated the third germ tube at 6 h, and 52.5% germinated the third germ tube at 8 h. While only 3.7% of the WT conidia germinated the second germ tube at 6 h, and 2.9% germinated the third germ tube at 8 h. While only 3.7% of the WT conidia germinated the second germ tube at 6 h, and 1.6% germinated the third germ tube at 8 h. These results indicate that deletion of the *plcH* gene leads to polar abnormality in *A. fumigatus*.

In fungi, activation of the CWI signaling pathway triggered by a cell wall defect involves transmitting the signal through an Rsr1, Cdc42, and Rho1 cascade, and the MAPK pathway is then activated through Pkc1 to regulate cell wall synthesis. Activation of Rho1 and Rho3 also induces activation of Bni1/SepA, which regulates actin cytoskeleton rearrangement and thus affects polarized growth^43^, ^44^, ^45^, ^46^ (Figure 6D). Additionally, kinesin KipA, Lag1, and Sur2 are also involved in polarity^47^, ^48^, ^49^, ^50^. The expression levels of these genes were analyzed (Figure 6E). In the early stage of germination, polarity was established after 4 h of incubation. At this time point, the expression of *rsr1*, *rho1*, *sepA*, and *kipA* was upregulated (Figure S4A). After incubation for 6 and 9 h, the expression of *rsr1*, *cdc42*, *rho1*, *rho3, bni1*/*sepA*, *kipA*, *lag1*, and *sur2* was downregulated (Figure S4B,C). These results suggest that activation of the CWI signaling pathway induces a polar abnormality of the mutant at the germination stage.

**Figure 7:**
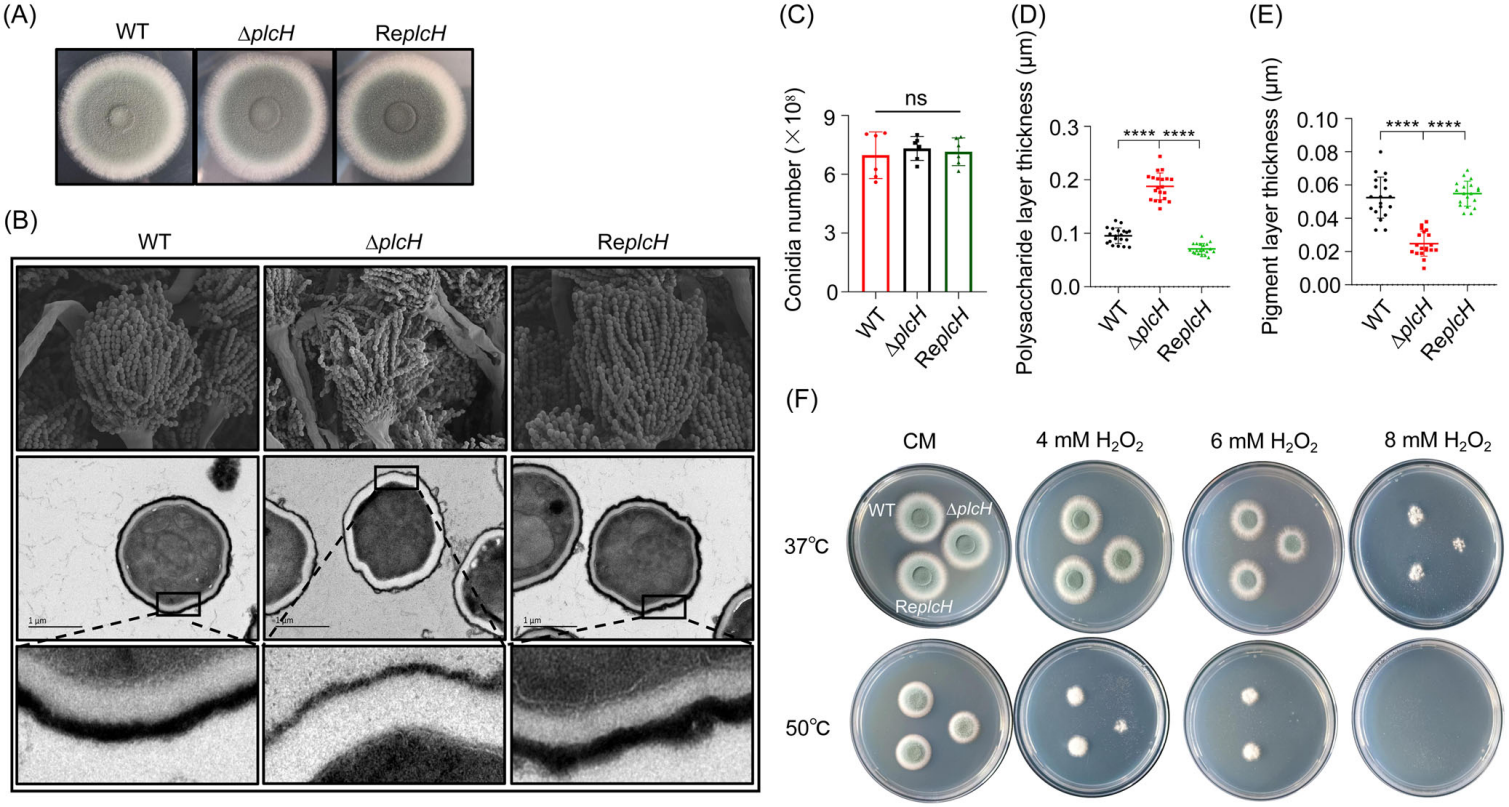
Conidial cell wall of the Δ*plcH* mutant. (A) Colony of the WT, the mutant, and the revertant strain. A total of 10^3^ freshly washed conidia from the WT, the Δ*plcH* mutant, and the revertant strain were spotted on solid CM plates and incubated at 37°C for 72 h. (B) Morphology of the mutant under electron microscopy. The conidia were fixed and examined using scanning electron microscopy (upper panel) and transmission electron microscopy (middle and lower panels) as described in the Materials and Methods section. (C) Conidia counting. The conidiation was statistically analyzed using a blood‐counting chamber. (D) Measurement of the conidial cell wall of the mutant. The thickness of the cell wall polysaccharide layer was measured using ImageJ software. (E) Measurement of the pigment layer of the conidial cell wall. The thickness of the outermost pigment layer of the cell wall was measured using ImageJ software. The results are presented as the mean ± SD. Asterisks show statistically significant differences (ns, not significant; ****p < 0.0001) based on one‐way ANOVA (WT vs. Δ*plcH* and Re*plcH* vs. Δ*plcH*). (F) Sensitivity of the mutant to H_2_O_2_. A total of 10^6^ freshly washed conidia from the WT, the Δ*plcH* mutant, and the revertant strain were spotted on solid CM plates containing 4, 6, and 8 mM H_2_O_2_, and incubated at 37°C and 50°C.

In comparison to the WT, the mutant showed a similar mycelial growth rate at 37°C but a significant decrease in the growth rate at 50°C (Figure S5A–D). Conidia counting revealed that the conidiation of the mutant was similar to that of the WT and the revertant (Figure 7A,C). Although the morphology of the mutant conidia appeared normal under scan electron microscopy (Figure 7B, upper panel), a thickened polysaccharide layer and a thinner pigment layer were observed under transmission electron microscopy (Figure 7B, middle panel and lower panel; Figure 7D,E). The pigment layer can protect conidia from the stimulation of reactive oxygen species and phagocytosis of macrophages^51^, ^52^, ^53^, ^54^. As shown in Figure 7F, the mutant conidia with thinner pigment layers were less resistant to H_2_O_2_ at 50°C. These results indicate that deletion of the *plcH* gene leads to a severe defect in the conidial cell wall of *A. fumigatus*.

### Deletion of the *plcH* gene leads to a reduced immune response

**Figure 8:**
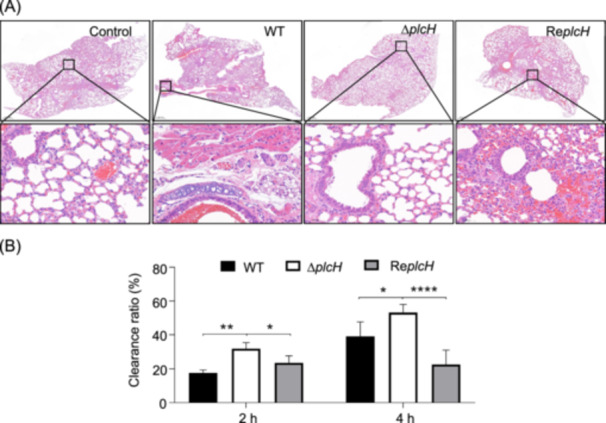
Immune response of the Δ*plcH* mutant in vivo and in vitro. (A) Virulence of the WT, the Δ*plcH* mutant, and the revertant strain detected with immunosuppressed mice and shown by hematoxylin‐eosin (HE) staining. The control group was inoculated with 0.01% Tween 20 in saline. The right lung from each mouse was dissected at day 3 postinfection and fixed in 4% (v/v) paraformaldehyde in physiological saline. (B) Survival rate of the mutant conidia in THP‐1‐derived macrophages. The survival of *A. fumigatus* conidia in the immune cells was determined after 2 h and 4 h of incubation of THP‐1‐derived macrophages with conidia. The results are represented as the mean ± SD from at least three independent experiments. Asterisks show statistically significant differences (*p < 0.05; **p < 0.01; ****p < 0.0001) based on one‐way analysis of variance (WT vs. Δ*plcH*, Re*plcH* vs. Δ*plcH*).

To evaluate the significance of PlcH in virulence, freshly harvested conidia of the WT, Δ*plcH*, and revertant were separately inoculated into immunocompromised mice. The mice were monitored continuously for 30 days after inoculation with conidia. No significant difference in virulence was observed between the Δ*plcH* mutant and the WT. As shown in Figure 8A, in the lung tissues of the mice inoculated with the WT or revertant conidia, the histology of the lesions was characterized by marked necrosis surrounded by numerous macrophages and neutrophils. However, necrosis and neutrophilic infiltration were much gentler in the lung tissues infected by the Δ*plcH* conidia, indicating a reduced immune response of the Δ*plcH* mutant. Macrophages can phagocytose and kill fungal conidia. We also determined the killing of conidia by macrophages. As shown in Figure 8B, at 2 and 4 h post incubation with THP‐1‐derived macrophages, the phagocytic ratio of the Δ*plcH* mutant was dramatically higher than that in the WT or the revertant, suggesting that deletion of the *plcH* gene results in faster clearance of conidia by macrophages.

## DISCUSSION

GPI‐APs produced by *A. fumigatus* are vital for the cell wall, polarized growth and thus the infection process^21^, ^22^, ^31^, ^32^, ^55^, ^56^. Some GPI‐CWPs, such as Mp1 and Mp2, have been identified as immunogenic proteins that induce host immune response^16^, ^19^. These GPI‐CWPs can serve as diagnostic biomarkers or potential targets of antifungal therapies. Therefore, it is vital to reveal how these GPI‐CWPs are secreted from the plasma membrane to the cell wall of *A. fumigatus*.

In yeasts, two GH76 family proteins, Dfg5 and Dcw1, are involved in the secretion of GPI‐CWPs into the cell wall^25^, ^26^, ^27^, ^28^. Although several members of the GH76 family have been found in filamentous fungi, such as *N. Crassa* and *A. fumigatus*, none are involved in the secretion of GPI‐CWPs^25^, which might be through a pathway different from that in yeasts. On the other hand, in mammalian cells, some GPI‐APs can be released from the cell membrane by GPI phospholipase to become secreted proteins. Thus, we hypothesized that the GPI‐phospholipase‐mediated release of GPI‐APs would be a possible pathway for the secretion of GPI‐CWPs in *A. fumigatus*.

In this study, a phospholipase gene, *plcH*, was identified from the *A. fumigatus* genome and successfully expressed in *E. coli*. An activity assay of the purified recombinant protein confirmed that PlcH is a phospholipase C that can specifically hydrolyze the phosphate ester bond between lipid and inositol to release GPI‐CWPs from the cell membrane. Mp1 is a specific GPI‐CWP covalently linked to β‐glucan of the cell wall^55^. Fusion of the C‐terminal sequence of Mp1, which contains the region from ω‐23 to ω‐1 and the GPI signal sequence, to the C‐terminus of GFP can direct cell wall localization of the chimeric protein GFP‐Mp1^31^. In this study, we adopted the same strategy to track the localization of GPI‐CWPs by expressing GFP‐Mp1 in either the WT or the mutant strain. As a result, the GFP‐Mp1 fusion protein was found in the cell membrane of the mutant but not in the cell wall of the WT, which demonstrates that PlcH can recognize a certain signal contained in the region from ω‐23 to ω‐1 of Mp1 and specifically release Mp1 from the plasma membrane of *A. fumigatus*.

Although it is known that GPI‐APs with basic amino acids at the ω‐1 or ω‐2 sites are ultimately localized to the cell membrane of *A. fumigatus* ^31^, the signal that directs the localization of GPI‐APs in the cell wall is still unknown. To identify the GPI‐CWPs released by PlcH in *A. fumigatus*, GPI‐CWPs were extracted from the cell walls of the WT and the mutant and analyzed using LC‐MS/MS. Twenty GPI‐CWPs were identified in the WT. No basic amino acid was found at the ω‐1 or ω‐2 position of these GPI‐CWPs, which is consistent with our previous finding^31^. Proteomic analysis of the mutant cell wall proteins revealed that seven GPI‐CWPs were missing in the conidial and mycelial cell wall. No basic amino acid residue was found in the region from ω‐1 to ω‐23 of these proteins, and this pattern was also shared by the six GPI‐APs released from the plasma membrane by recombinant PlcH, suggesting that PlcH can specifically catalyze the cleavage of GPI‐APs without any basic amino acid in the region from ω‐1 to ω‐23. However, it remains unknown how the GPI‐CWPs containing basic amino acids at the region from ω‐1 to ω‐23 are released.

The *A. fumigatus* cell wall can be divided into two sublayers: the outer and inner layers. The outer layer includes α1,3‐glucan, glycoproteins, galactomannan, mannan, melanin, and galactosaminogalactan. The inner layer is composed of β1,3‐glucan and chitin covalently linked to other polysaccharides, such as β1,6‐glucan and galactomannan. The outer layer α1,3‐glucan is synthesized by Ags1‐Ags3, while the inner layer β1,3‐glucan is synthesized by Fsk1, Gel1‐Gel7, and Bgt1‐Bgt3. We previously showed that inhibition of the intact GPI‐anchor synthesis results in a significant reduction in cell wall glycoprotein and β‐glucan in *A. fumigatus*, which leads to cell wall defects^21^. In this study, we also found that glycoproteins and β‐glucans were significantly reduced, whereas α‐glucans were significantly increased in the mutant strain. The cell wall defect in the mutant activated the CWI signaling pathway, which then induced the altered expression of the genes involved in the synthesis of β‐glucan and α‐glucan. As a result, the synthesis of α‐glucan was upregulated to compensate for the reduction of cell wall proteins and β‐glucan. These results suggest that PlcH‐related assembly of the cell wall glycoproteins is crucial for CWI in *A. fumigatus*.

Although seven GPI‐APs were missing in the mutant and six GPI‐APs could be cleaved by recombinant PlcH, only three GPI‐APs overlapped in both assays, including cell wall galactomannoprotein Mp1 (EAL84472.1), cell wall galactomannoprotein Mp2 (EAL87607.1), and a conserved hypothetical protein (EAL87073.1). The difference between the in vivo and in vitro assays is probably due to the difficulty of completely removing noncovalently linked proteins in the network of cell wall polysaccharides or nonspecific cleavage by recombinant PlcH. However, undoubtedly Mp1, Mp2, and EAL87073.1 can be specifically released by PlcH from the membrane of *A. fumigatus*.

*A. fumigatus* infection is initiated with the inhalation of conidia. When dormant conidia come into contact with airway epithelial cells, they start to germinate and invade patients' lung tissue. Dormant conidia are covered with a hydrophobic rodlet layer and a pigment layer. Both the rodlet layer and the pigment layer can protect conidia from recognition by the host immune cells and thus escape alveolar macrophage killing^19^. At the germination stage, cell wall proteins, glucans, galactomannan, and chitin are exposed to alveolar macrophages to induce or suppress an immune response^57^. In our study, we did not observe any change in virulence when the mutant conidia were tested in the immunocompromised mice; however, a reduced inflammatory response in the mice infected with the mutant and increased killing of the mutant conidia by macrophages were observed. As β1,3‐glucan can be recognized by Dectin‐1 and induce inflammation^58^, the weak inflammation response induced by the mutant can be ascribed to the decrease in β1,3‐glucan in the mutant. On the other hand, the pigment layer serves as a shield to avoid stimulating reactive oxygen species and enables conidia to escape phagocytosis and damage by macrophages^51^, ^52^, ^53^, ^54^. We consistently showed that the mutant was more sensitive to H_2_O_2_. Therefore, it can be concluded that increased macrophage killing of the mutant is due to the decrease in pigment.

In conclusion, for the first time, we show that PlcH can specifically release some GPI‐CWPs from the plasma membrane of *A. fumigatus*, which represents a newly discovered secretory pathway of GPI‐CWPs in *A. fumigatus*. A lack of PlcH leads to defects in CWI, conidiation, polarity, and increased sensitivity to antifungal drugs. Although only a few proteins are identified as GPI‐CWPs released by PlcH, a lack of these GPI‐CWPs results in an attenuated inflammatory response in an immunocompromised mouse model and increased macrophage killing. Our results suggest an important role for these GPI‐CWPs in the interaction of *A. fumigatus* with host cells. Our findings not only provide a deeper understanding of the mechanism of cell wall synthesis but also provide new targets for diagnosis and antifungal therapies.

## MATERIALS AND METHODS

### Strains and culture conditions

*A. fumigatus* KU80 (*pyrG* ^*−*^) was used as the WT strain to construct the mutant strains (Table S4). *A. fumigatus* strains were grown on complete medium (CM) or minimal medium (MM) using 0.5 mM sodium glutamate as the sole nitrogen source^59^. 5 mM uridine and uracil were added for the *pyrG^−^*‐genotype strains. Conidia were harvested from solid CM by washed with 0.1% Tween 20 and measured by hemocytometer counting.

### Expression and purification of fusion protein

TBLASTN search was performed using the PLC sequences of *C. orthopsilosis*, *C. albicans*, *B. thuringiensis*, and *S. aureus*. A 1434‐bp open reading frame (AFUA_4G12000), renamed *plcH* gene, was retrieved from the *A. fumigatus* genome database. The *plcH* gene was amplified from *A. fumigatus* cDNA using primer pair P1 and P2 (Table S5) and cloned into the pGEM‐T easy vector (Promega) to generate T‐PlcH. The cDNA of the *plcH* gene was then amplified from T‐PlcH and subcloned into pGEX‐6P‐1 (HonorGene) to construct the recombinant plasmid pGEX‐PlcH. *E. coli* BL21(DE3) harboring pGEX‐PlcH was incubated in 100 ml Luria‐Bertani (LB) medium containing 50 μg ampicillin ml^−1^ at 37°C. A final concentration of 0.4 mM of IPTG was added to the culture medium when the OD_600_ value reached 0.6. After induction at 16°C overnight, the cell extracts were prepared and separated by GST‐tag Purification Resin (Beyotime). The recombinant protein was confirmed by SDS‐PAGE and MS. Protein concentration was determined by the Bradford assay^60^.

### Phospholipase C activity assay

Phospholipase activity was determined with phospholipids as substrates. A total volume of 200 µl of reaction mixture consisted of 50 mM Tris‐HCl (pH 8.0), 1 mM dithiothreitol, and 100 µg of various substrates. Upon the addition of 50 µg purified protein, the reaction mixture was incubated at 30°C for 1 h. The reaction product was extracted using 2% orthophosphoric acid and chloroform‐methanol (2:1). Products in the organic phase were analyzed by TLC using chloroform/methanol/28% ammonia (65:25:10) as solvent^61^.

### Assay for the activity of PlcH toward GPI‐anchor

Fresh conidia were inoculated into liquid CM and cultivated at 37°C for 48 h. Mycelia were collected, ground in liquid nitrogen, and resuspended in 0.05 M Tris‐HCl (pH 7.8). The cell wall and membrane were separated by centrifugation at 13,000 rpm for 15 min. The supernatant was further ultra‐centrifuged (125,000*g*) to obtain microsomes. Microsomes were resuspended in 20 mM Tris‐HCl containing 2 mM EDTA (pH 8.0) and ultra‐centrifuged at 50,000*g* to obtain a clean cell membrane fraction. The membrane pellets were incubated with or without 50 µg of the purified PlcH at 30°C for 2 h. Activity assay was carried out in reaction buffer (50 mM Tris‐HCl containing 0.14 M NaCl and 2 mM CaCl_2_, pH 7.4). The reaction mixture was ultracentrifuged at 50,000*g* for 40 min. After the incubation, the released proteins were collected by ultracentrifugation (50,000*g*) for 40 min and treated with NH_3_·H_2_O followed by HNO_2_ to release inositol 1‐phosphate. Proteins were removed with chloroform: n‐butanol (3:1). After spinning dry, the extracted derivative was dissolved in double distilled water and analyzed by HPLC.

### Construction of the mutant and revertant strain

To construct the *plcH‐*deletion mutant, the deletion cassette containing *pyrG* gene was generated to replace the coding region of *plcH* ^62^. The 1.3‐kb upstream region of the *plcH* before the ATG start codon was amplified using the primer pair P3 and P4, and the 1.5‐kb downstream region of the *plcH* after the stop codon was amplified using the primer pair P5 and P6 (Table S5). The PCR products were digested with *Not*I/*Sma*I/*Nde*I and cloned into the relevant sites of pGEM‐T. By the digestion of pCDA14 (a gift from the Institut Pasteur) with *Hpa*I, the 8.4‐kb *pyrG* blaster cassette was obtained and cloned into the *Hpa*I site between the upstream and downstream of the *plcH* to yield pGEM‐koPlcH.

To construct the revertant strain, the *plcH* gene (1.4‐kb) with its upstream region (1.3‐kb) was amplified using primer pair P7 and P8 (Table S5). The downstream fragment (1.5‐kb) of *plcH* gene was amplified using primer pair P9 and P10 (Table S5). The PCR products were cloned into pGEM‐T to generate pGEM‐H. The 3.6‐kb *pyrG* cassette was obtained by *Xba*I‐digestion of pCDA14 and cloned into pGEM‐H to yield pGEM‐PlcH. The resulting plasmid was introduced into *A. fumigatus* KU80 (*pyrG^−^*) as reported previously^63^. The identification of positive transformants were confirmed using PCR and Southern blot with the 1‐kb upstream flanking region serving as a probe. The probe was labeled using a DIG‐labeled hybridization kit (Roche Applied Science).

### Microscopy and fluorescence

Plasmid *GpdA*‐Pro::*chiB1*‐N‐signal‐*gfp*‐*Mp1*‐C contains *GpdA* promoter, a *chiB1* signal peptide sequence and subcellular localization signal sequence of the *A. fumigatus mp1* gene, synthetic *gfp*(2‐5) gene of GFP from plasmid pMCB17, and *trpC* terminator sequence (EMBLZ32690) from *A. nidulans* ^64^. The PCDA14 plasmid containing *pyrG* and the plasmid containing *gfp‐mp1* gene were transformed into *A. fumigatus* KU80 (*pyrG^−^*) and the ∆*plcH* mutant. The WT and the ∆*plcH* mutant strains harboring *GpdA*‐Pro::*chiB1*‐N‐signal‐*gfp*‐*Mp1*‐*trpC* were cultured on CM at 37°C for 48 h to examine the subcellular localization of chimeric GFP‐Mp1, respectively. Mycelia were subjected to plasmolysis by 0.5 M sorbitol for fluorescence microscopy (Leica Microsystems)^31^.

### Protein extraction and western blot analysis

Freshly collected conidia were cultured at 37°C. Mycelia were obtained using paper filtration, followed by rinsing with distilled water and subsequent storage at −80°C for further use. The membrane fraction and the cell wall were isolated as in previous reports^65^, ^66^. The mycelia were ground in liquid nitrogen by hand. The lyophilized powder was dissolved in 200 mM Tris‐HCl (pH 7.8) containing 20 mM EDTA and 0.1% Protease Inhibitor Cocktail at 4°C overnight for thorough lysis. The cell wall was prepared by centrifugation (10,000*g*) at 4°C for 10 min and boiled three times in 50 mM Tris‐HCl (pH7.8) containing 2% SDS, 20 mM sodium EDTA, and 40 mM β‐mercaptoethanol to remove noncovalently linked proteins. The supernatant of the dissolved lyophilized powder was subjected to ultracentrifugation (125,000*g*) at 4°C for 1 h to collect membrane pellets. The membrane pellets were resuspended in 20 mM Tris‐HCl (pH 8.0) containing 2 mM EDTA and 0.1% Protease Inhibitor Cocktail, homogenized, and then centrifuged at 125,000*g* at 4°C for 1 h. This process was repeated three times to remove nonmembrane components. One milligram of dried cell wall was treated with 10 μl of hydrofluoride (HF)‐pyridine at 0°C for 3 h^67^. After centrifugation, proteins extracted by HF were precipitated with 9 volumes of 100% methanol buffer and detected by SDS‐PAGE and western blot^31^.

### LC‐MS/MS analysis

Proteins extracted from *A. fumigatus* and *E. coli* BL21(DE3) were separated using SDS‐PAGE, followed by staining with Coomassie brilliant blue. Subsequently, each protein band on the gel was isolated and submitted to the MS detection platform at the Institute of Microbiology, Chinese Academy of Sciences for identification^68^.

### Phenotypic analysis of the mutant

10^5^ conidia were incubated on solid CM or in liquid CM at 37°C or 50°C. The growth rate was measured by determining colony diameter and biomass. Conidia were grown on solid CM at 37°C or 50°C and collected by water containing 0.01% Tween for counting.

A serial dilution of conidia of the WT, Δ*plbH*, and revertant strains from 10^6^ to 10^3^ was spotted on the CM with Congo red and Calcofluor white, and incubated at 37°C for 24 h or 50°C for 36 h.

A series of 10‐fold dilutions (10^6^–10^3^ cells) of *A. fumigatus* conidia were spotted on solid CM containing 2 μg/ml Itraconazole, 0.4 μg/ml Clotrimazole, 1 μg/ml Terbinafine, and 30 μg/ml Amphotericin, respectively. After incubation at 37°C or 50°C for 36 h, the plates were photographed. Strain KU80 (*PyrG* ^*+*^) was used as a control.

### Chemical analysis of cell wall components

10^8^ conidia of *A. fumigatus* were incubated in liquid CM at 37°C for 48 h with shaking (200 rpm). Mycelia were collected by filtration, washed twice with distilled water, and lyophilized. Ten milligrams of dry mycelia were ground in 50 mM NH_4_HCO_3_ (pH 8.0) using Disruptor Genie (Scientific Industries) with 0.2 g of glass beads (0.5 mm diameter) for 1 h. The cell wall pellets were collected and treated with 1 M KOH at 70°C for 0.5 h to release alkali‐soluble glycoproteins and α‐glucans. When the pH of alkali‐soluble materials was adjusted to 5.0, α‐glucans were precipitated and collected by centrifugation. Glycoproteins in the supernatant were precipitated with 4 volumes of 100% ethanol and dissolved in distilled water. Protein was determined by Lowry protein assay^66^.

### Microscopic analysis

To analyze the structure of *∆plcH* strains, 10^8^ conidia were spread on solid CM and liquid CM, and then incubated at 37°C for 48 h. Conidia and mycelium were examined by Scanning Electron Microscopy (FESEM SU8010, HITACHI). Ultrastructure was examined with Transmission Electron Microscopy (FEI Company)^22^, ^69^.

10^6^ conidia were inoculated into 10 ml liquid CM with glass coverslips and incubated at 37°C for 5–8 h. At the intervals, the germlings adhering to the coverslip were examined with a microscope.

### RNA extraction and qRT‐PCR

Total RNA of *A. fumigatus* was isolated using the TRIZOL method (Invitrogen). cDNA was synthesized with the TIANGEN FastKing RT kit (TIANGEN). The quantitative real‐time RT‐PCR (qRT‐PCR) was performed using 2×SYBR® Green Pro Taq HS Premix Ⅲ (Accurate Biology). The *TBP* gene encoding the TATA‐box binding protein was used as an internal control. Each sample was tested in triplicate. The primers used in this assay are listed in Table S5.

### Virulence test

Virulence of the WT, ∆*plcH*, and revertant strains was detected with immunosuppressed mice as the standard protocol described previously^21^, ^52^, ^70^, ^71^. Mice were injected with cyclophosphamide (150 mg/kg mouse weight) on Days −3, −1, +3, +6, and +9, and hydrocortisone (200 mg/kg mouse weight) on Day −1. Fresh conidia of the WT, ∆*plcH*, and Re*plcH* were suspended in 0.01% Tween 20 in saline; 30 ml of conidia (3 × 10^5^ CFU/g mouse weight) were inoculated to each immunosuppressed mouse by nasal feeding on Day 0. Ten male BALB/c mice (18–20 g) were used in each group; 0.01% Tween 20 in saline was used as a control. After inoculation, mice were monitored twice every day and mortality was recorded. Mice survived on Day 30 were humanely terminated. Three days after intranasal injection, three mice were randomly selected for dissection, and their right lungs were excised and fixed in 4% (v/v) paraformaldehyde saline. Sections were stained with hematoxylin‐eosin (HE) and periodic acid‐Schiff (PAS) by standard techniques.^69^

### Measurement of conidial phagocytosis

THP‑1 (American Type Culture Collection) was cultured in RPMI 1640 (Gibco) containing 10% FBS (Gibco) at 37°C with 5% CO_2_. THP‑1‑derived macrophages were prepared by incubation of THP‑1 cells with 100 ng/ml of PMA (cat. no. P1585, Sigma‑Aldrich) for 24 h. Adherent macrophages were maintained in a CM at 37°C with 5% CO_2_ for 48 h. *A. fumigatus* resting conidia (MOI = 1) were incubated with THP‑1‐derived macrophages for 2 h. Macrophage killing assay was performed as previously described^72^, ^73^. Briefly, nonphagocytosed conidia and non‐adherent cells were washed away with pre‐warmed PBS three times. The cells were subsequently incubated for 0 (control), 2, and 4 h in a fresh medium. The cells were lysed by freezing at −80°C and thawing at 37°C. Conidia in the cell lysate were diluted with distilled water, inoculated on MM agar, and incubated at 37°C for 36 h. Colonies on MM plates were counted and compared with control (0 h).

## AUTHOR CONTRIBUTIONS

**Jinbin Hao**: Data curation (equal); formal analysis (equal); investigation (equal); software (equal); validation (equal); writing—original draft (equal). **Yin Guo**: Investigation (supporting); methodology (supporting); resources (supporting). **Hui Zhou**: Investigation (supporting); methodology (supporting). **Haomiao Ouyang**: Methodology (equal); resources (equal). **Jinghua Yang**: Project administration (supporting). **Wenxia Fang**: Data curation (equal); methodology (equal); project administration (equal); resources (equal); supervision (equal); validation (equal). **Cheng Jin**: Conceptualization (equal); data curation (equal); funding acquisition (equal); project administration (equal); supervision (equal); writing—review and editing (equal).

## ETHICS STATEMENT

This study was approved by the Ethics Committee of the Institute of Microbiology of Chinese Academy of Sciences (Permit number: SQIMCAS2021).

## CONFLICT OF INTERESTS

The authors declare no conflict of interests.

## Supporting information

Supporting information.

## Data Availability

All the data are available in the main text and Supporting Information.
