# Supplementary material for: Phospholipase PlcH is involved in the secretion of cell wall glycoproteins and contributes to the host immune response of Aspergillus fumigatus
Source: mLife. 2024 Dec 26;3(4):537–50. doi: 10.1002/mlf2.12146 (PMC11685838; doi:10.1002/mlf2.12146)
Supplement: Supplementary file 1 — Supporting information. [file MLF2-3-537-s001.PDF]

Supporting information

Phospholipase PlcH involves in secretion of cell wall glycoproteins and contributes to host immune response of *Aspergillus fumigatus*

Jinbin Hao[1,2], Yin Guo[3], Hui Zhou[1], Haomiao Ouyang[1], Jinghua Yang[1], Wenxia Fang[4]*, Cheng Jin[1,2]*

[1] State Key Laboratory of Mycology, Institute of Microbiology, Chinese Academy of Sciences, Beijing 100101, China

[2] University of Chinese Academy of Sciences, Beijing, China

[3] Colleg of Life Science and Technology, Guangxi University, Nanning, China

[4] Institute of Biological Sciences and Technology, Guangxi Academy of Sciences, Nanning, China

TABLE S1 Thirteen GPI-CWPs were found in both WT and Δ*plcH* mutant.

Uniprot ID	Predicted GPI protein	Function	Subcellular Localization
P0C957.1	Yes	Lysophospholipase 1	cell wall, Extracell
EAL91643.1	Yes	conidial hydrophobin Hyp1/RodA	cell wall
P0C958.1	Yes	Lysophospholipase 3	cell wall, Extracell
EAL85466.1	Yes	hydrophobin, putative	cell wall
EAL89828.1	Yes	GPI anchored protein, putative	cell wall
Q4WCX9.1	Yes	GPI-anchored protein	cell wall, Extracell
Q4WDN4.1	Yes	Probable aspartic-type endopeptidase OpsB	cell wall, Extracell
Q4WEP7.1	Yes	Chitinase A1	cell wall, Extracell
Q4WG16.1	Yes	Probable glucan endo-1,3-beta-glucosidase EglC	cell wall
P41746.2	Yes	Hydrophobin	cell wall
Q4WLB9.1	Yes	GPI-anchored CFEM domainprotein A	cell wall
Q4WNV0.1	Yes	Aspartic-type endopeptidase CtsD	cell wall, Extracell
Q8X176.1	Yes	Acid phosphatase PhoA	cell wall, Extracell

TABLE S2 GPI-APs disappeared in the cell wall of the Δ*plcH* mutant.

Uniprot ID	Predicted GPI protein	Function	Subcellular Localization
EAL84472.1	Yes	cell wall galactomannoprotein Mp1	cell wall
EAL84834.1	Yes	conserved hypothetical protein	cell wall
EAL92862.1	Yes	WSC domain protein, putative	cell wall, Extracell
EAL87073.1	Yes	conserved hypothetical protein	cell wall, Extracell
EAL87607.1	Yes	cell wall galactomannoprotein Mp2	cell wall
EAL92273.1	Yes	extracellular serine-threonine rich protein	cell wall, Extracell
EAL84649.2	Yes	conserved glycine-rich protein	cell wall, Extracell

TABLE S3 GPI-APs released from cell membrane by purified PlcH.

Uniprot ID	Predicted GPI protein	Function	Subcellular Localization
Q4WDN4.1	Yes	Probable aspartic-type endopeptidase OpsB	cell wall, Extracell
EAL84472.1	Yes	cell wall galactomannoprotein Mp1	cell wall
EAL87073.1	Yes	conserved hypothetical protein	cell wall, Extracell
EAL84834.1	Yes	conserved hypothetical protein	cell wall
EAL87607.1	Yes	cell wall galactomannoprotein Mp2	cell wall
EAL92273.1	Yes	extracellular serine-threonine rich protein	cell wall, Extracell

TABLE S4 *A. fumigatus* strains used in this study.

Strain name	Genotype	Reference
WT	*ku80; pyrG*	Cove 1966
WT^{ko}	*ku80; ΔpyrG*	Cove 1966
Δ*plcH*	*ku80; ΔpyrG*; *ΔplcH::neo-pyrG-neo*	This study
Δ*plcH*^{neo}	*ku80*; *ΔplcH::neo*	This study
Re*plcH*	*ku80*; *ΔplcH::neo::plcH::pyrG*	This study
WT/*GFP-Mp1*	*ku80; ΔpyrG::GpdA*-Pro*::chiB1*-N-signal*::gfp::Mp1*-C*::pyrG*	This study
Δ*plcH*/*GFP-Mp1*	*ku80; ΔpyrG*; *ΔplcH::neo-pyrG-neo::GpdA*-Pro*::chiB1*-N-signal*::gfp:: Mp1*-C*::pyrG*	This study

TABLE S5 Primers used in this study.

Primer name	Sequence 5'-3'
For protein expression	
P1	ATGGTGGCCGAACACTTGACGATTCG
P2	TTAGTGTTCCTGCTCCTCTCTCATCTG
For deletion	
P3	ATAAGAATGCGGCCGCGGACTTCGGTGATCCCTGCGTAG
P4	TCCCCCGGGGTTAACTGTGCGACAAGCCAGTGCTCAATT
P5	TCCCCCGGGGTTAACCGACGCTTGATTTGTGCTTTCACG
P6	GGGAATTCCATATGGGTTATCGGCGCCAACCATTG
PyrG F	TTCCTAATACCGCCTAGTC
PyrG R	AATCACCTCAATCACACCA
plcH F	TGGCCGAACACTTGACGATTC
plcH R	ATCTTTGGATGGGTCCTCTGGG
For complementation	
P7	ATAAGAATGCGGCCGCGGACTTCGGTGATCCCTGCGTAG
P8	GCTCTAGATTAGTGTTCCTGCTCCTCTCTCAT
P9	GCTCTAGACGACGCTTGATTTGTGCTTTCACG
P10	GGGAATTCCATATGGGTTATCGGCGCCAACCATTG
For qRT-PCR	
rsr1 F	GTTGCCTTACTGCTCAATTCGTT
rsr1 R	TCTGCTTTCGATAGGAGTCTTCAAT
cdc42 F	GGAGCTGGGTGCTGTAAAATACGTC
cdc42 R	GCCGCAACAATCGCCTCATC
rho1 F	TTTCATACCCCGACTCCCACG
rho1 R	CGGAGATCCACTTCTCCTGG
rho3 F	ATACCTTGAATGCTCTGCTG
rho3 R	TTCACATCAAGAGCGACCTT
sepA F	TGGTGGAGTGGAAGAAATCGAAAG
sepA R	GCCGGCGTTGACACGTTTGC

kipA F	CTATTTATATGACAATGTCTTCCCGC
kipA R	CTTCTTACAAGGCGCTTGGC
sur2 F	GGCTTTACGTCACTTTCCACTCTCG
sur2 R	AAGCCTTCCACCGGGTGATT
lag1 F	GTTGAGTTACCGGTTCCACT
lag1 R	TTTTTGAGGTCGCTAGGAAG
swoC F	AACTATGACAAGCTCATGGTGAGG
swoC R	TTCAGGTCACGCTTGAGAGG
TBP F	CCACCTTGCAAAACATTGTT
TBP R	TACTCTGCATTTCGCGCATG
ags1 F	GTCGCTACTTTGGACTACTTACAGG
ags1 R	ATGCTGATCCAAAAGCGTTGTATCC
ags2 F	CTTCAAGGAATGGGCATCAA
ags2 R	GATCGTGCCTGCCAGATAGA
ags3 F	ACTTTCGAGCATGTACTGAATTCCA
ags3 R	ATCAAACCTGTTCCAGCAAAGTAGA
bgt1 F	ATATCATCCCTGCGGCTAAG
bgt1 R	GTCGGTGAAAGATTTGTCGT
bgt2 F	GCAATACCAAGTCCGACGG
bgt2 R	TGACAAGGGCATGAACCAT
fsk1 F	GGAACGCGAACTTGAGAGGA
fsk1 R	AAGGGCAGAGTACAAACGGG
gel1 F	GATGGTTGCAAGCGTGAC
gel1 R	CATCGTGGTTCTTGGAGTTG
gel2 F	AGTCTCAACCACGACGAGTG
gel2 R	AGGTATCCGCCATACAAAGG
gel3 F	TTGGATACGCGACCACTGAC
gel3 R	TTGTCACCGCACCATTCGTA
gel4 F	AACTCCGACTACACCGATCC
gel4 R	AGGTACGGATGACATTGGTG
gel5 F	TCTGACCCATCCCAGTCGAT
gel5 R	GTTGGAAACCTCATTGCCCG
gel6 F	CGTATCAGCTCGTTCCCGAT
gel6 R	GCCAGAACTTTCATGCAGCC
gel7 F	TACTACGAGCAACAGTCCGC
gel7 R	GGAAATGCCAGCCTCCTTCA

The underlined sequences are the engineered restriction cut sites for DNA cloning; F, forward; R, reverse.

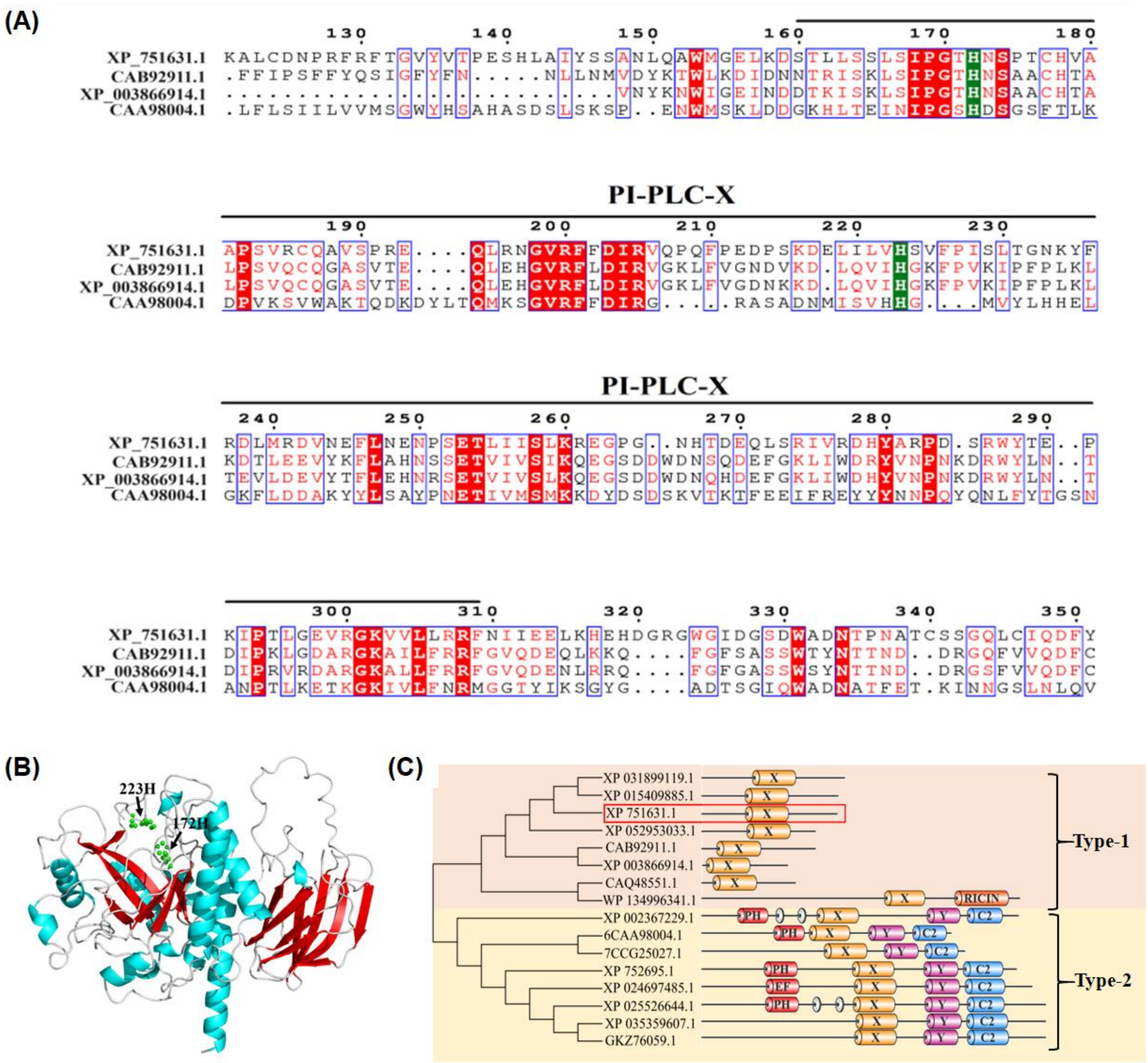

FIG S1 Alignment, simulation and phylogenetic analysis of PlcH. (A) Multiple alignment of the amino acid sequence of PlcH orthologues from different fungal species. The conserved residues are highlighted in red and the catalytic amino acids His-172 and His-223 are highlighted in green. PI-PLC-X domain is indicated in black line. (B) Simulation of 3-D structure of PlcH. SWISS-MODEL was used for homology modeling and PyMOL was used for tertiary structure visualization. (C) The phylogenetic tree of the PlcH homologs was constructed by neighborjoining method using MEGA 6.0.

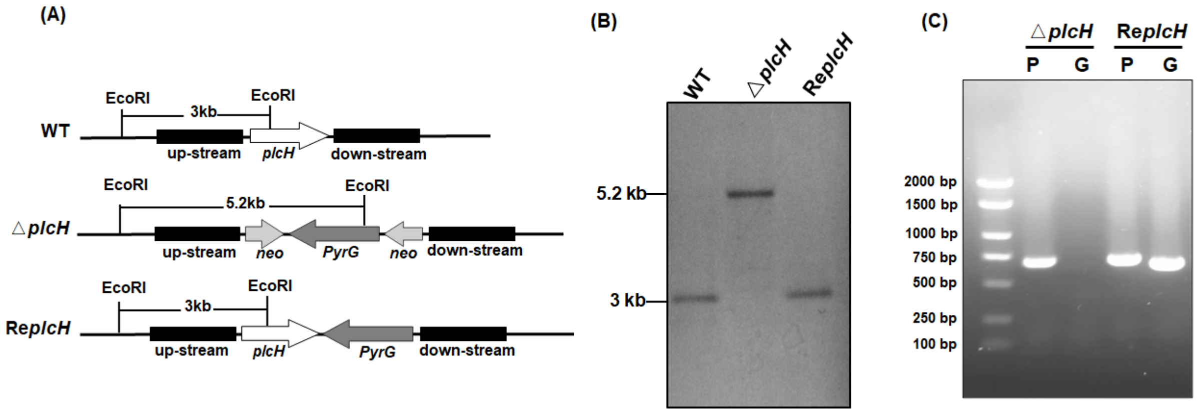

FIG S2 Construction of the mutant and revertant of *plcH* gene. (A) Schematic diagram of the WT, mutant and revertant. (B) Confirmation of the mutant and revertant by Southern blot. Genomic DNA digested with *EcoR* I was probed with a 1 kb up-stream of the *plbH* gene. A 3.0-kb fragment was detected in the WT and revertant strains, while a 5.2-kb fragment was detected in the Δ*plcH* mutant. The electrophoretic positions and sizes of DNA are indicated in Southern blotting. (C) Confirmation of the mutant and revertant by PCR. The null mutant Δ*plcH* was constructed by replacing of the *plcH* gene with *pyrG* and the revertant strain Re*plcH* was constructed by introducing of the *plcH* gene into the mutant as described in Materials and Methods. In (C), P, *pyrG*; G, *plcH*. The *pyrG* gene band could be amplified but *plcH* gene band could not be amplified in the Δ*plcH* mutant by PCR, while both *pyrG* and *plcH* genes could be amplified in the revertant strain.

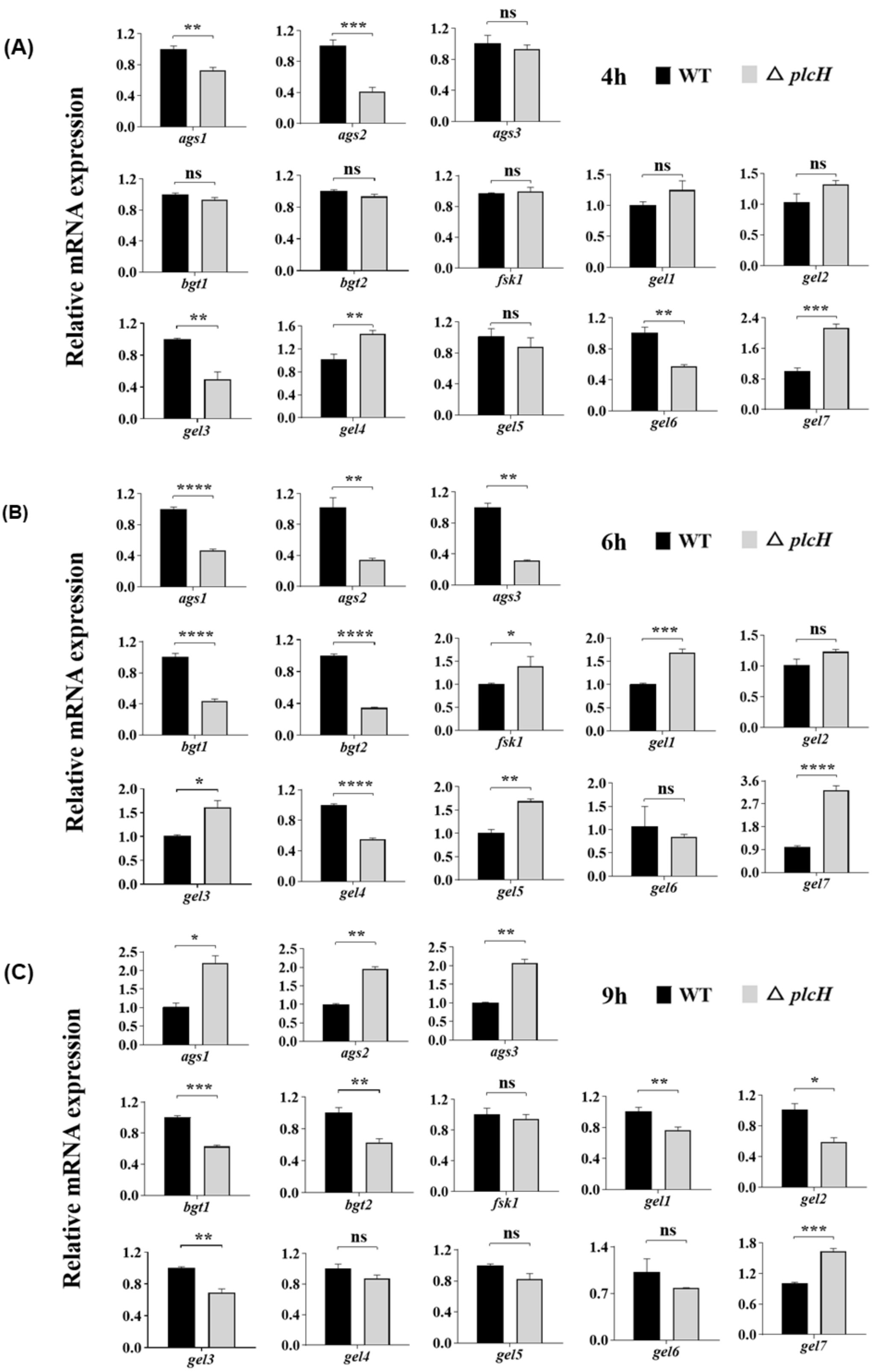

FIG S3 Expression of the genes responsible for cell wall polysaccharide synthesis during germination of the Δ*plcH* mutant. Gene expression was detected at 4 h (A), 6 h (B) and 9 h (C) after spore liquid culture.

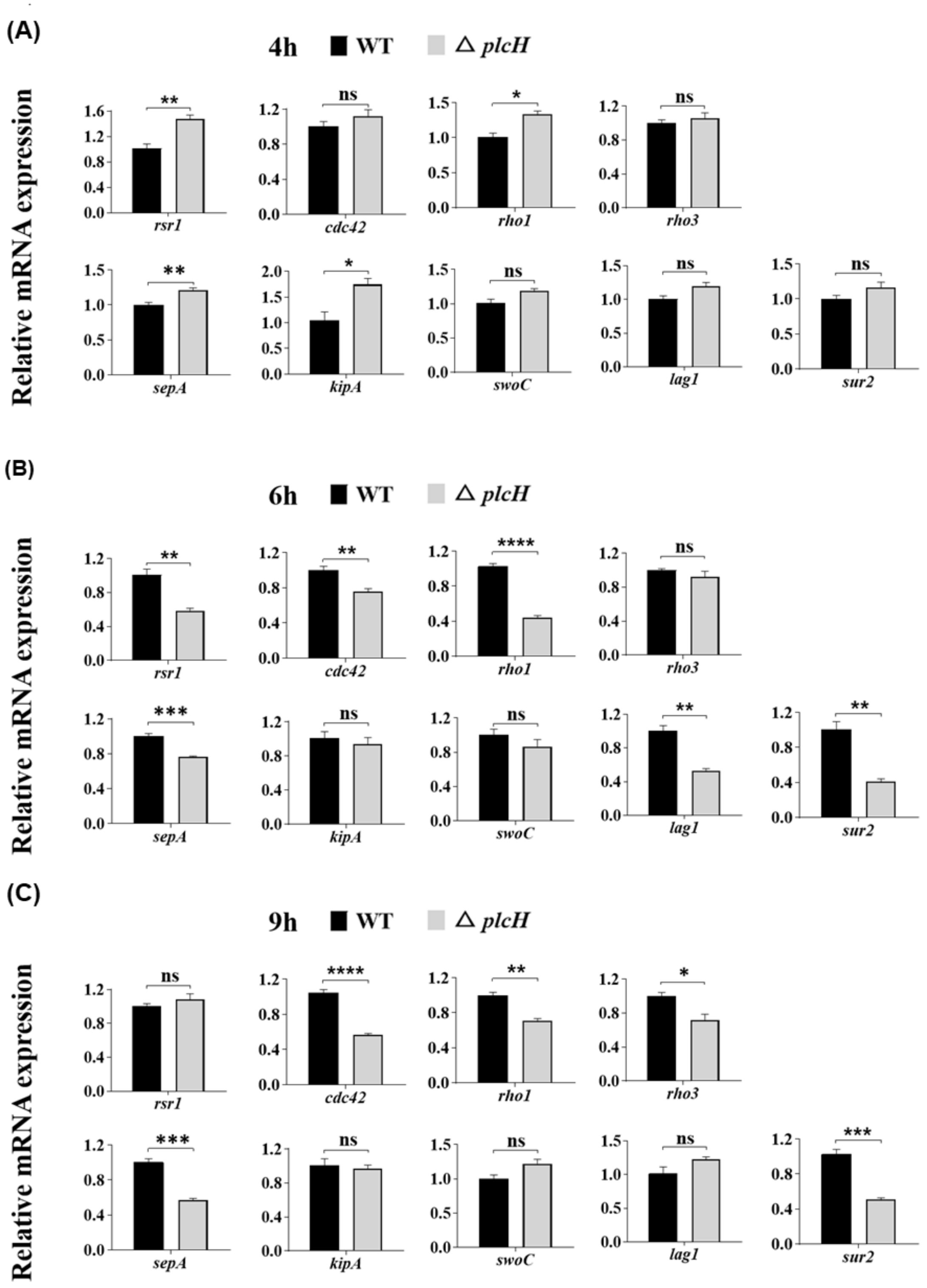

FIG S4 Expression of the genes related to cell polarity during germination of the Δ*plcH* mutant. Gene expression was detected at 4 h (A), 6 h (B) and 9 h (C) after spore liquid culture.

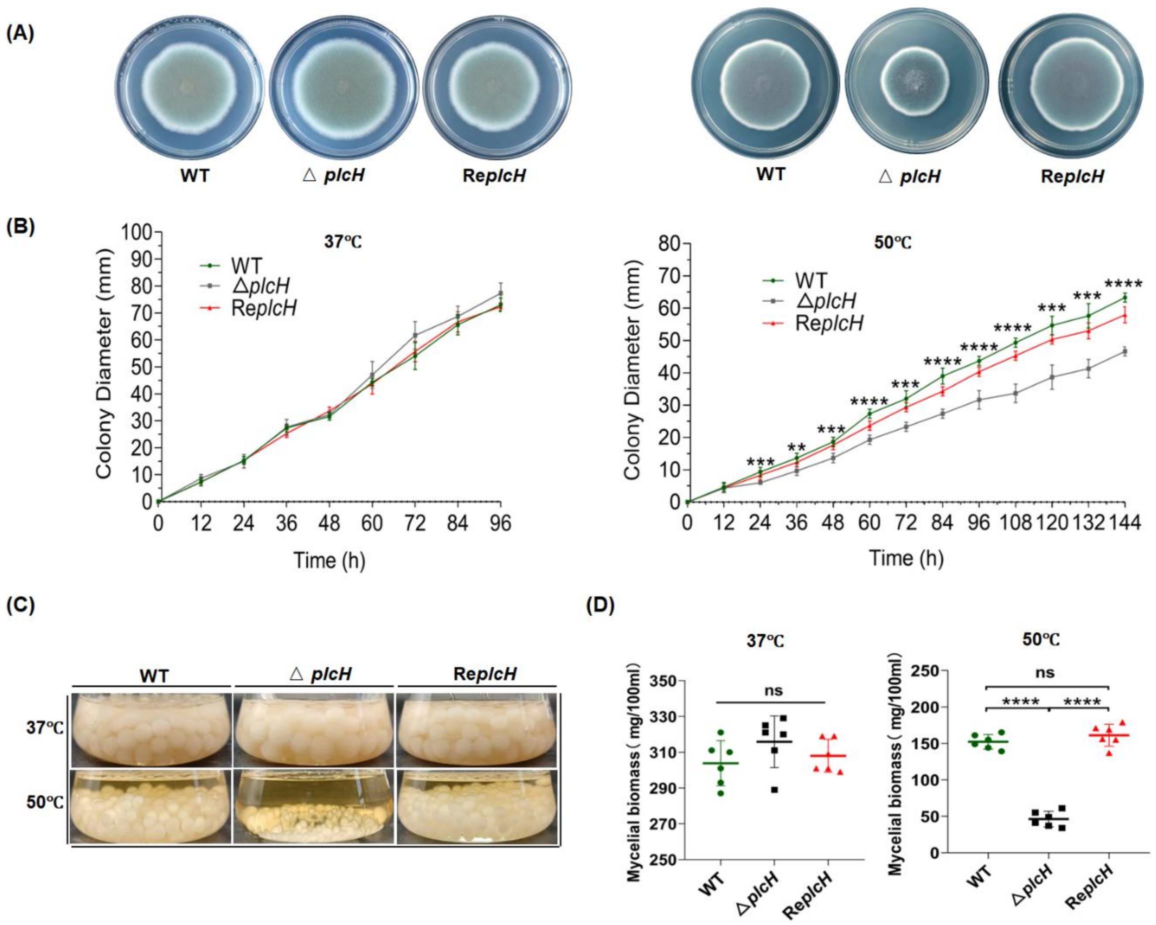

FIG S5 Hyphal growth of the Δ*plcH* mutant. (A and B) Growth rate of the WT, mutant and revertant strains on solid CM. 10^3 freshly washed conidia from the WT, Δ*plcH* mutant and revertant strains were spotted on solid CM plates and incubated at 37℃ for 96 h and 50℃ for 144 h. Colony diameters were measured at the corresponding time points and averaged. Growth kinetics was plotted using the mean diameter. (C and D) Growth rate of the WT, mutant and revertant strains in liquid CM. 10^3 freshly washed conidia from the WT, Δ*plcH* mutant, and revertant strains were inoculated into 100 mL of liquid CM and incubated at 37 ℃ and 50 ℃. After 48 h of incubation, the mycelia were collected by filtration and then lyophilized and weighed, and the results were shown in D. The Results are representative of six independent experiments ± SD. ns, not significant; ****$p < 0.0001$.
